# High prevalence of undiagnosed chronic kidney disease among at-risk population in Kinshasa, the Democratic Republic of Congo

**DOI:** 10.1186/1471-2369-10-18

**Published:** 2009-07-21

**Authors:** Ernest K Sumaili, Eric P Cohen, Chantal V Zinga, Jean-Marie Krzesinski, Nestor M Pakasa, Nazaire M Nseka

**Affiliations:** 1 Nephrology Unit, University of Kinshasa, Kinshasa, PO BOX 123 KIN XI, Democratic Republic of Congo; 2 Nephrology Division, Medical College of Wisconsin, 9200 W Wisconsin Ave, Milwaukee, WI, 53226, USA; 3 Division of Nephrology/Transplantation, University of Liege, Sart Tilman B35, PO BOX 4000 LIEGE 1, Belgium; 4 Previous address: Department of Pathology, University of Kinshasa, Kinshasa, Democratic Republic of Congo

## Abstract

**Background:**

There is limited knowledge of Chronic Kidney Disease (CKD) among high risk populations, especially in the developing countries. We report our study of testing for CKD in at-risk subjects.

**Methods:**

In a cross-sectional study, 527 people from primary and secondary health care areas in the city of Kinshasa were studied from a random sample of at-risk out-patients with hypertension, diabetes, obesity, or HIV+. We measured blood pressure (BP), blood glucose level, proteinuria, body mass index, and estimated glomerular filtration rate (eGFR by MDRD equation) using calibrated creatinine levels based on one random measurement. The associations between health characteristics, indicators of kidney damage (proteinuria) and kidney function (<60 ml/min/1.73 m^2^) were also examined.

**Results:**

The prevalence of CKD in this study was 36%, but only 12% were aware of their condition. 4% of patients had stage 1 CKD, 6% stage 2, 18% stage 3, 2% stage 4, and 6% had stage 5. 24 hour quantitative proteinuria (>300 mg/day) was found in 19%. In those with the at-risk conditions, the % of CKD was: 44% in patients with hypertension, 39% in those with diabetes; 16% in the obese and 12% in those who were HIV+. 82% of those with a history of diabetes had elevated serum glucose levels at screening (≥ 126 mg/dl). Only 6% of individuals with hypertension having CKD had reduced BP to lower than 130/80 mmHg. In multivariate analysis, diabetes, proteinuria and hypertension were the strongest determinants of CKD 3+.

**Conclusion:**

It appears that one out of three people in this at-risk population has undiagnosed CKD and poorly controlled CKD risk factors. This growing problem poses clear challenges to this developing country. Therefore, CKD should be addressed through the development of multidisciplinary teams and improved communication between traditional health care givers and nephrology services. Attention to CKD risk factors must become a priority.

## Background

Chronic Kidney Disease (CKD) is a worldwide health problem [1]. Indeed, the incidence and prevalence of CKD has increased in recent years in both developed and developing countries [2] including in Sub-Saharan Africa (SSA) [3]. In SSA, CKD affects mainly young adults in their productive years and is a significant cause of death [3,4]. This may occur by death from kidney failure, and also by cardiovascular deaths, which are increased in those with CKD. Major contributory factors for this ominous picture include late referral to hospital, limited renal replacement therapy (RRT), limited capacity of health workers for CKD detection and prevention, and poor awareness of kidney disease in the community [3,5,6].

This situation prompted the International Society of Nephrology Commission for the Global Advancement of Nephrology (ISN COMGAN) to make the fight against CKD one of its priorities, by promoting awareness, early detection, and effective treatment [7].

In our recent survey [4] in the general population of Kinshasa, the capital of the Democratic Republic of Congo (DRC) the prevalence of early stage CKD was 10 times greater than that of stage 5 CKD or end stage renal disease (ESRD). Thus, while severe CKD can result in progressive kidney failure, the effects of less-severe CKD are quantitatively more important. Although cumulative evidence shows that early detection and treatment prevents or delays some of its adverse outcomes [8-11], the majority of subjects at early stages of CKD are undiagnosed and under-treated in SSA [6]. Indeed, because of the scarcity of financial resources and shortage of manpower, CKD prevention programs are either rudimentary or virtually nonexistent in the developing world [12].

Furthermore, attention to health problems in SSA is usually been focused on infectious diseases, rather than on chronic non-communicable disease (CND) such as CKD.

Screening of specific groups for CKD, such as those with known diabetes (DM) or with hypertension (HTN), may be more cost effective than screening the general population [13]. But, the benefits of targeted screening of patients with DM and HTN versus screening of general population are debatable [14]. A non validated Modification of Diet in Renal Disease (MDRD) study equation in Black Africans, would probably lead to an over or underestimation of CKD prevalence in Sub-Saharan population.

Nonetheless, since we found an increasing prevalence of CKD risk factors such as HTN, DM, and obesity in Kinshasa [4], along with the co-occurrence of human immunodeficiency virus (HIV) infection and HIV-associated renal diseases [15], it seemed reasonable to target screening on these groups [16]. The objective of this survey was to experiment early detection of a large numbers of previously unidentified persons with or at high risk for CKD. In addition, we determined the prevalence of CKD across selected risk factors and according to the level of kidney function within groups. Finally, socio-demographic features and clinical parameters of this at-risk group were assessed.

## Methods

### Study design and population

The present cross-sectional study is the second part of a larger ongoing study of CKD and associated risk factors termed "Prévalence, détection précoce et prévention des maladies rénales chroniques et facteurs de risque associés (PDMRA) en République Démocratique du Congo ". The present survey was done between January 9 and May 25, 2007.

Screening sites included primary (n = 9) and secondary health care (n = 4) chosen at random among networks of healthcare areas existing in the city of Kinshasa. The choice of the networks was based on the following criteria: a high number of the patients at-risk of CKD attending the centre, agreement of the authorities and availability of the health workers in these centres to select the subjects as well as to accommodate the research workers. PDMRA screening was conducted by a mobile research team composed of nephrologists, nurses and laboratory technicians. Primary care is a term used to define the activity of a health care provider who acts as a first point of consultation for all patients, while secondary health care includes dispensation of the care, hospital admissions and quality control of the structures of primary care.

Eligibility criteria for screening were age of 18 years or older and a personal history of HTN, DM, obesity or HIV infection, or first degree relative with kidney disease.

### Screening Examinations and data collection

Knowing that 12.4% of adults have CKD in Kinshasa [4], approximately 473 subjects were required to reach that prevalence with error estimate of 3% [17]. Of an intended sample size of 550, 527 persons (291 in primary care and 236 in secondary care) agreed to participate. But, for three of them, the sample of serum was not properly handled.

All participants provided informed written consent before enrolment. They were examined by the research team, which recorded information on demographics, diet, smoking, alcohol consumption, use of indigenous herbal drugs, and birth weight knowledge. Data about first degree family relatives and medical history for kidney disease, HTN, DM and current treatment were also recorded. Body weight, height and waist circumference were measured. HTN, DM, obesity and HIV infection had been diagnosed during past patient visits. These diagnoses were confirmed in writing. All HIV participants except twelve received standard weight-based dosages of highly active antiretroviral therapy (HAART), but medications were not adjusted for kidney function. They were initiated on stavudine plus lamivudine with either nevirapine (82%) or efavirenz (4%).

Blood pressure (BP) was measured twice in the right or left arm using a calibrated sphygmomanometer (WelchAllyn, Germany) at heart level. The subjects were allowed to relax for 5 minutes in a sitting position before determination of blood pressure.

HTN was defined as a systolic blood pressure ≥ 140 mmHg or diastolic blood pressure ≥ 90 mmHg and/or concomitant use of antihypertensive medications by self-report [18].

BP was categorized according to the Seventh Joint National Committee Report on Detection, Evaluation and Treatment of High Blood Pressure [18]. The categories were as follows:

- normal, < 120 and < 80 mmHg,

- prehypertension, 120–139 or 80–89 mm Hg;

- stage 1, 140–159 or 90–99 mm Hg;

- stage 2, ≥ 160 or ≥ 100 mm Hg.

The body mass index was calculated from the measured weight (in kilograms) and height (in meters) and was categorized as not obese (< 25 kg/m^2^), overweight (25 to 29.9 kg/m^2^) or obese (≥ 30 kg/m^2^) according to the 2000 WHO criteria [19].

The diagnosis of DM was established after two fasting glucose values of ≥ 126 mg/dl using fingertip blood (Accutrend glucometer) and/or concomitant use of antidiabetic medications. DM was classified as type 2 or type 1 by clinical features as follows. Illness starting after age 40 years, the presence of chronic complications at onset, and an absence of ketones determined the diagnosis of type 2 diabetes. On the other hand, illness onset before or at age 30, and typical clinical signs (weight loss, asthenia, polyuria, polydipsia) determined the diagnosis of type 1 diabetes.

The participants provided a urine sample to detect proteins by urinary strips '(Combur 7-test)". Female subjects were instructed to void a random urine specimen, remote from menstrual periods. If positive with dipstick protein of 3+ (n = 102), measurement of 24-hour urinary protein was obtained (n = 99). Because ratio of albumin to creatinine (ACR) was not available and because urine dipstick provides only a semi-quantitative estimation of proteinuria and has imperfect accuracy in diagnosis of persistent proteinuria, kidney damage in stage 1 and 2 CKD in our study was identified as 24-hour urinary protein ≥ 300 mg per day. Serum creatinine and 24-hour quantitative proteinuria were carried out according to the kinetic Jaffe (semi-automated polyphotometer Visual Biomérieux) and Esbach methods, respectively. These tests were performed in the laboratory of the Belgian medical centre of Kinshasa ''CMK". For estimated Glomerular Filtration Rate (eGFR) determination, the abbreviated equation from the MDRD study was used. We calibrated the creatinine results measured with the Jaffe method against a traceable isotope dilution mass spectrometry (IDMS) enzymatic method (creatinine +, Roche enzymatic diagnostics) as described [4]. Recalibrated serum creatinine values were thereafter computed for each participant and the new MDRD study equation was used for estimation of the eGFR as 175 × (serum creatinine level [(mg/dl)])^-1.154 ^× (age [(years)])^-0.203 ^[20]. For women and for blacks (all patients in our study), the product of this equation was multiplied by correction factor of 0.742 and 1.21, respectively.

The K/DOQI guidelines [21] for definition and classification of CKD were used in the present study. The CKD stages are defined as follows: stage 1, proteinuria ≥ 300 mg per day with an eGFR higher than 90 ml/min/1.73 m^2^; stage 2, proteinuria ≥ 300 mg per day with an eGFR of 60 to 89 ml/min/1.73 m^2^; stage 3, an eGFR of 30 to 59 ml/min/1.73 m^2^; stage 4, an eGFR of 15 to 29 ml/min/1.73 m^2^; and stage 5, an eGFR <15 ml/min/1.73 m^2^.

The term chronic renal failure or CKD 3+ refers to an eGFR< 60 ml/min/1.73 m^2^. The term "all stages of CKD" includes both kidney damage (early stage of CKD, 1 and 2) and chronic renal failure (CKD 3 or worse).

### Statistical analysis

Results are presented as number and percentage or mean ± SD. 2-sample Student's t test and chi-squares were used for comparison of means and proportions, where appropriate.

The outcome under analysis was the presence of CKD, defined as above. Exposure variables that were considered included gender, age, smoking, herbal drugs use, BMI, DM, pulse pressure, family history of kidney disease (FH-KD), HTN, and HIV infection. The crude (unadjusted) relationships between the exposure variables and the presence or absence of CKD were examined in univariate logistic regression analyses. Multivariate stepwise logistic regression analysis was then done to evaluate the simultaneous effects of various exposure variables, with adjustment for the potential confounding effects of other factors. The above approaches were applied separately for CKD stage 3+ and for proteinuria.

A multiple linear regression model was used to determine the independent association between the reduction of an eGFR < 60 ml/min/1.73 m^2 ^and continuous variables such as age, duration of DM, duration of HTN, BP, and BMI. Except chi-squares, all data analysis and calculations were performed by using a standard statistical package (SPSS Inc, Chicago, IL version 13.0, 2004). Chi-squares were made in the dialog box using medcalc version 9.1.01, 2005. A *P *value < 0.05 indicated statistical significance. All rules of confidentiality were complied with, including collection of information and physical examination. This study was approved by ethics committee of the Provincial Medical Inspection of Kinshasa.

## Results

### A. Characteristics of the study population

Sociodemographic characteristics of the participants are listed in Table 1. The age range was between 18–90 years old (mean 54 ± 15, median 55). Some statistically significant differences between male and female subjects were observed.

**Table 1:** Population characteristics.

Clinical features	Males N = 229	Females N = 298	Total N = 527	*P*
Age mean ± SD (yr)	55.3 ± 15.3	52.8 ± 14.9	53.9 ± 15.5	0.05
Age range n (%)				
-18 to 43 yr	53 (23.1)	83 (27.9)	136 (25.8)	0.25
-44 to 55 yr	52 (22.7)	83 (27.9)	135 (25.6)	0.20
-56 to 67 yr	69 (30.1)	78 (26.2)	147 (27.9)	0.37
-> 67 yr	55 (24.0)	54 (18.1)	109 (20.7)	0.12
Hypertension n (%)	129 (56.3)	175 (58.7)	304 (58.2)	0.62
Diabetes n (%)	125 (54.5)	162 (54.4)	287 (54.5)	0.76
- Type 1	8 (6.4)	6 (3.7)	14 (4.9)	0.4
- Type 2	117 (93.6)	156 (96.3)	273 (95.1)	0.4
Obesity n (%)	14 (6.1)	71 (23.8)	85 (16.1)	< 0.0001
HIV infection* n (%)	22 (32.4)	60 (49.6)	82 (43.4)	0.03
FH-KD first degree* n (%)	13 (7. 3)	25 (11.7)	38 (9.7)	0.12
History of Kidney disease n (%)	15 (6.8)	9 (3.1)	24 (4.6)	0.07
Smoking currently n (%)	28 (12.3)	10 (3.4)	38 (7.3)	0.0002
Herbal remedy use n (%)	58 (25.4)	37 (12.5)	95 (18.2)	0.0002
Low educational level**	65 (29.0)	162 (55.5)	227 (44.0)	< 0.0001

Values expressed as numbers and proportions in parentheses or mean ± SD, as appropriate.

SD, standard deviation. yr, years.

Percentages based on total participant number except for * HIV infection status or responding number.

** low level education (< 6 years).

### B. CKD according to health care level

The overall prevalence of CKD stage 1–5 was 36% (95% CI, 36.0 to 36.4) (Table 2) and was higher in males than in females, being 40% versus 34%, respectively. Of these 190 subjects with CKD, 14% had reduced eGFR MDRD equation < 60 ml/min/1.73 m^2 ^(CKD stage 3 or more). However, only twenty four of those with CKD (12%) were aware of their condition. 24 h quantitative proteinuria ≥ 300 mg was detected in 19% of the studied population. Although all stages of CKD were found in both levels of health care, 9 patients (3%) and 24 patients (8%) in primary care and, 13 patients (6%) and 8 patients (3%) in secondary care had CKD stage 1 or 2, respectively. Thus, CKD stage 2 was more prevalent in primary care compared to secondary care (*P *< 0.05). In contrast, CKD stage 4 and 5 was more frequent in secondary health care than it was in primary care (*P *< 0.001). Most of patients with stage 5 CKD died quickly from uraemia because of lack of dialysis.

**Table 2:** Prevalence of CKD among at-risk population by level of health care.

Stages of CKD/Heath care (eGFR. ml/min/1.73 m^**2**^)	Primary N = 291 (%)	Secondary N = 236 (%)	Total N = 527 (%)	*P*
1(≥ 90)*	9 (3.1)	13 (5.5)	22 (4.2)	0.25

2 (60 to 89)*	24 (8.3)	8 (3.4)	32 (6.1)	0.03

3a (45 to 59.9)	43 (14.9)	21 (8.9)	64 (12.2)	0.05

3b (30 to 44.9)	23 (8.0)	9 (3.8)	32 (6.1)	0.07

4 (15 to 29.9)	1 (0.3)	9 (3.8)	10 (1.9)	0.007

5 (< 15)	2 (0.7)	28 (11.9)	30 (5.7)	< 0.0001

All stages of CKD	102 (35.3)	88 (37.4)	190 (36.2)	0.68

Proteinuria ≥ 300 mg/day	52 (17.9)	47 (19.9)	99 (18.7)	0.6

Values expressed as numbers and proportions in parentheses or mean ± SD, as appropriate.

Percentages based on responding number or total participant number. For three patients, the sample of serum was not properly handled (missing data).

* 24-hours urinary protein excretion ≥ 300 mg/day. eGFR based on MDRD study.

CKD is defined as either kidney damage (proteinuria ≥ 300 mg/day) and/or kidney function (< 60 ml/min/1.73 m^2^).

**Figure 1:**
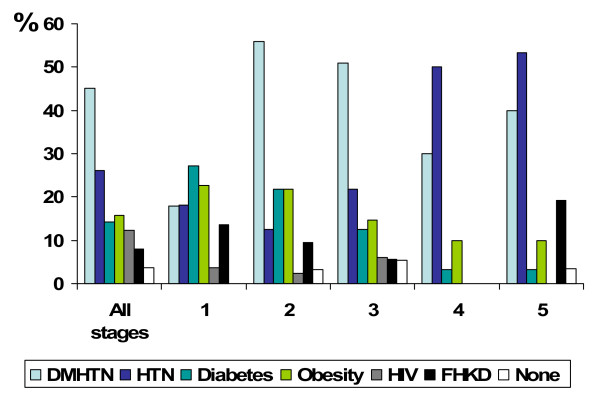
**Prevalence (%) of CKD among selected populations by stage of CKD**. Abbreviations: DMHTN = patients with diabetes and hypertension, HTN = hypertensive, HIV+ = person having human immunodeficiency virus infection antibody positive, FHKD = family history of kidney disease; CKD = chronic kidney disease. CKD is defined either kidney damage (proteinuria ≥ 300 mg/day) or kidney function (eGFR < 60 ml/min/1.73 m^2^).

In addition, the prevalence of CKD among subjects at-risk according to K/DOQI stage is shown in figure 1. In those with the at-risk conditions, the prevalence of CKD was: 45% in patients with DM and HTN, 26% in patients with HTN, 16% in the obese and 12% in those who were HIV+. In persons having FH-KD, CKD was found in 8%. Table 3 shows the 24-hour quantitative urine protein, dipstick abnormalities in the urine (proteinuria, pyuria and hematuria) and elevated creatinine (men > 1.6 mg/dl and women > 1.4 mg/dl) according to K/DOQI stage.

**Table 3:** Prevalence of abnormal urinary findings and serum creatinine by K/DOQI stage of CKD.

Abnormalities/eGFR	< 15 N = 27 (%)	15–29 N = 10 (%)	30–59 N = 96 (%)	60–89* N = 32 (%)	≥90* N = 22 (%)
Dipstick proteinuria	25 (92.6)	8 (88.9)	81 (85.3)	32 (100.0)	22 (100.0)
Proteinuria ≥ 300 mg/day	18 (66.7)	3 (33.3)	24 (25.3)	32 (100.0)	22 (100.0)
Dipstick hematuria	9 (33.3)	3 (33.3)	11 (11.6)	7 (34.3)	3 (13.6)
Dipstick pyuria	4 (14.8)	4 (44.4)	58 (61.0)	21 (65.6)	10 (45.4)
S. creatinine (men >1.6 md/dl/women > 1.4 mg/dl	27 (100.0)	10 (100.0)	53 (55.2)	1 (3.1)	0

Values expressed as numbers and proportions in parentheses or mean ± SD, as appropriate.

* 24-hours urinary protein excretion ≥ 300 mg/day. eGFR based on MDRD study.

CKD is defined either as kidney damage (proteinuria ≥ 300 mg/day) and/or kidney function (< 60 ml/min/1.73 m^2^).

Percentages based on total number of dipstick proteinuria available (n = 521).

S. creatinine = serum creatinine; SD, standard deviation.

In the 82 people with HIV, quantitative proteinuria at more than 300 mg/day was observed in five subjects; five others had CKD stage 3+.

### C. Hypertension and/or Diabetes, and CKD status

Table 4 gives BP control according to JNC VII [18] and serum glucose levels of the participants according to their CKD status. CKD patients were much more likely to have HTN than those without CKD (*P *= 0.0005). 82% of participants with history of DM had elevated fasting serum glucose levels at screening (≥ 126 mg/dl [≥ 7 mmol/l]), and 78% of those with history of HTN did not have their BP controlled to less than 140/90 mmHg. Among individuals with HTN having CKD, 99% received treatment. However, only 6% of all individuals with HTN had their BP reduced to lower than 130/80 mmHg and 22% had BP lower than 140/90 mmHg. The majority of the treated individuals with HTN were receiving one antihypertensive medication and, only 23% used Angiotensin-Converting Enzyme Inhibitors (ACEI).

**Table 4:** Hypertension and/or diabetes, and CKD status.

Clinical features	CKD patients	Non CKD patients	Total	*P*
**JNC 7 guidelines** **All patients (BP in mmHg)**				
Normal, < 120 and < 80	52 (27.5)	143 (43.2)	195 (35.5)	0.0005
PreHBP, 120–139 or 80–89	32 (16.9)	56 (19.6)	88 (16.9)	0.5
Stage 1, 140–159 or 90–99	38 (20.1)	72 (21.8)	110 (21.2)	0.7
Stage 2, ≥ 160 or ≥ 100	67 (35.4)	60 (18.1)	127 (24.4)	<0.0001
**Hypertensives**				
Antihypertensive drugs				
none	1 (1.1)	11 (10.6)	22 (11.2)	0.01
Any treatment	89 (98.9)	93 (89.4)	182 (88.8)	0.01
1 antihypertensive	62 (71.3)	60 (57.7)	122 (62.2)	0.06
mean antihypertensive drugs	1.28 ± 0.5	1.22 ± 0.6	1.25 ± 0.6	0.1
ACEI*	21 (23.3)	33 (31.7)	54 (27.7)	0.2
Normal, < 120 and < 80	7 (5.2)	17 (10.0)	24 (7.9)	0.1
High normal, 120–139 or 80–89	22 (16.4)	21 (12.4)	43 (14.1)	0.4
Stage 1, 140–159 or 90–99	38 (28.4)	72 (42.4)	110 (36.2)	0.01
Stage 2, ≥ 160 or ≥ 110	67 (50.0)	60 (35.3)	127 (41.8)	0.01
< 130/80	8 (6.0)	21 (12.4)	29 (9.5)	0.09
< 125/75	4 (3.0)	11 (6.5)	15 (4.9)	0.2
Serum glucose level (mg/dl)				
**Diabetics**				
< 110	14 (12.8)	17 (9.8)	31 (11.0)	0.5
110–125	8 (7.3)	12 (6.9)	20 (7.1)	0.9
≥ 126	87 (79.8)	145 (83.3)	232 (82.0)	0.5
≥ 180	45 (41.3)	91 (52.3)	136 (48.1)	0.09

Values expressed as numbers and proportions in parentheses or mean ± SD, as appropriate.

CKD is defined either kidney damage (proteinuria ≥ 300 mg/day) and/or kidney function (< 60 ml/min/1.73 m^2^).

Percentages based on responding number, not total participant number.

ACEI: Angiotensin-Converting Enzyme Inhibitors. PreHTN = Prehypertension.

Furthermore, only 4% of patients with DM and HTN having CKD had BP controlled to less than 130/80 mmHg and ACEI treatment was prescribed in only 29%. Patients with DM were treated with insulin (53%), oral medication such as metformin or glibenclamide (41%) or insulin combined with oral antidiabetic medication (1%). Table 5 summarizes the current problems of CKD, ESRD, and cost.

**Table 5:** Projected Annual cost arterial hypertension, Diabetes and ESRD Treatment.

Medications	Cost per patient (US$)	Total cost of all patients projected (US$)
Antihypertensive		
Thiazide diuretics	100	280 million
Calcium blocker	250	700 million
ACEI	1000	2.8 billion
Antidiabetic		
Biguanide-Metformin	200	240 million
Insulin	350	420 million
Renal replacement treatment		
Peritoneal dialysis	36,000	~1.4 billion*
Hemodialysis	63,000	~2.5 billion*

* Projection was made from about 1% of patients with diabetes or hypertension developing ESRD

ACEI = angiotensin-converting enzyme inhibitors.

ESRD = end stage renal disease

### D. Determinants of CKD

Determinants of CKD 3+ are displayed in Table 6. HTN (adjusted OR 3.3, 95% CI 1.7–6.5; *P *< 0.001), DM (adjusted OR 2, 95% CI 1.1–3.8; *P *= 0.01) and dipstick proteinuria (adjusted OR 2, 95% CI 1.1–3.6; *P *= 0.02) were independent factors associated with CKD 3+.

**Table 6:** Risk factors associated with CKD.

Determinants	OR (univariate analysis)	CI 95%	*P*	OR (multivariate analysis)	CI 95%	*P*
**CKD stage ≥ 3**						
						
HTN versus no	2.9	1.9 – 4.6	0.001	3.3	1.7 – 6.5	0.001
Pulse pressure > 60 versus < 60 mmHg	2.0	1.3–3.2	0.001	1.09	0.6 – 1.8	0.7
Age ≥ 50 versus <50 years	1.8	1.2–2.7	0.002	1.5	0.9–2.4	0.08
DM versus no	1.5	1.04–2.3	0.04	2.0	1.1–3.8	0.01
Dipstick proteinuria ≥ 1+ versus 0	2.9	1.7–5.1	< 0.0001	2.0	1.1–3.6	0.02
Herbal remedy use versus no	1.7	1.03–2.7	0.002	1.2	0.7–1.9	0.3
						
**24 h- quantitative proteinuria (≥ 300 mg/24 h)**						
						
Pulse pressure > 60 versus < 60 mmHg	2.4	1.5–3.9	< 0.0001	1.8	1.03 – 3.2	0.03
HTN versus no	1.7	1.1–2.8	0.01	1.2	0.7 – 2.2	0.4
DM versus no	1.7	1.05–2.8	0.03	1.4	0.8–2.4	0.1

Abbreviations: CKD 3+ (eGFR < 60 ml/min/1.73 m^2^), CKD = chronic kidney disease, CI = confidence interval, DM = diabetes, HTN= hypertension.

However, only pulse pressure > 60 mmHg (adjusted OR 1.8, 95% CI 1.03–3.2; *P *= 0.03) was statistically associated with the presence of proteinuria in this study.

In patients with DM, increasing age and duration of DM were associated with reduction of kidney function (as eGFR MDRD equation < 60 ml/min/1.73 m^2^) in multiple linear regression models according to the following equation:

[eGFR MDRD study equation = 61.6 - 0.12 (age, in years) - 0.43 (diabetes duration, in years), *P *= 0.03]. By contrast, in patients with HTN, no risk factors were associated with low eGFR in multiple linear regression models.

## Discussion

This study documents CKD among at-risk population in the health system of Kinshasa, a large city in Sub-Saharan Africa. The overall prevalence of undiagnosed CKD is high, at 36%. This prevalence is probably not real since it was based on non validated MDRD study equation. However, we think that the question of the validity of eGFR MDRD in Black race is debatable. Indeed, since the MDRD Study formula has been adequately validated in African Americans with kidney disease [22], extrapolation to Black Africans may be justified in view of similar genetic and physical attributes. But, validation should still be carried out. Nevertheless, the value of 36% is almost triple the prevalence of CKD in the general population of this city [4]. Similar trend has been noted in the Kidney Early Evaluation Program (KEEP) study [23], mainly for CKD 3+. This fact supports the use of targeted screening in identifying large numbers of subjects at-risk for CKD. It also confirms that the risk factors for CKD that are encountered in developed countries are also found in developing Africa.

In addition, our survey indicates that one person out of five in this group has proteinuria, which was occurred at all stages of CKD. Among individuals classified as having CKD stage 3, 25% had macroproteinuria. As some authors [14,24,25] have suggested, those subjects are the groups at most risk for cardiovascular disease (CVD) and CKD progression. With proteinuria and eGFR measurements, we have identify individuals with CKD stage 1 (4% of studied population) and CKD stage 2 (6%). In fact, identifying individuals with these earlier stages is of utmost importance since strong evidence emerge that the renal and cardiovascular risks that are associated with stage 1 and 2 are nearly equal to those of stage 3 [14]. Regrettably, in spite of this prevalence of proteinuria, in most cases it was neither detected earlier nor correctly managed before this study was done. Indeed, in almost all centres, 24 hour-quantitative proteinuria or dipstick proteinuria were not routinely available outside this study. Qualitative proteinuria was carried out in usual practice using the acetic acid method, which has limits of sensitivity and specificity [26]. Furthermore, and until now, in ordinary secondary care in Kinshasa, renal complications are assessed only by serum creatinine alone instead of resorting to formula -based estimates of creatinine clearance or eGFR. Consequently, many cases of CKD could be missed by using the serum creatinine alone.

In this present study, we found a discrepancy between prevalence of dipstick positive protein and proteinuria mainly in CKD stage 3. This difference may be due to the fact that only patients with dipstick positive protein 3+ were tested for 24-hour proteinuria. It also confirms differences in sensitivity and specificity between tests as reported recently by Konta et al. [27]. In their survey, the authors have demonstrated that dipstick positive proteins are more indicative of microalbuminuria than macroalbuminuria.

However, despite the fact that dipstick proteinuria has limited diagnostic value, it is of a great prognostic value. Indeed, dipstick positive protein has been associated with increased risk of cardiovascular events, including the development of HTN [28], DM [29], and ESRD [30]. But not all studies are in agreement [31] and it is unclear whether microalbuminuria is a marker of kidney disease or generalized vascular disease [32].

As expected [33], HTN, DM and proteinuria were independently associated with CKD 3+. Inadequate BP and/or poor glucose control may explain the CKD that we observed. The lack of BP control shown in our patients with HTN, of whom 78% were uncontrolled, is a bit higher than the 73% seen in Americans [34]. In addition, only 6% of participants with HTN and 4% of patients with DM and HTN having CKD had their BP controlled to the JNC VII recommended level of less than 130/80 mmHg [18]. This level of control is lower than the 20% in the New Opportunities for Early Renal Intervention by Computerised Assessment study (NEOERICA) [35] and the 11% reported in the National Health and Nutrition Examination Survey (NHANES III) [34]. It highlights the inadequate levels of BP control in this screening population, placing them at risk for cardiovascular and/or renal events, particularly ESRD [8] and may thereby increase medical care costs. Only 23% of all studied population received treatment to block the renin-angiotensin system. Certainly, ACEI may be less effective in Blacks, in whom the incidence of high renin hypertension is lower than in Caucasian population. However, the African-American Study of Kidney Disease and Hypertension (AASK) confirmed the reno-protective effects of ACEI compared to calcium blocker in Blacks with hypertensive nephropathy [36]. Also, in this AASK trial [36], an average of 2.6 drugs was needed to achieve BP goals, while the average number of drugs used by patients with HTN in the present survey was 1.3.

However, a secondary analysis of AASK study [37] comparing ACEI versus β blocker and Calcium blocker versus β blocker have shown that BP control does not always prevent progression of renal failure. These findings suggest that factors other than BP elevation likely participate in the progression of ''hypertensive" nephrosclerosis. But, we note that in this study [37], the authors did not test the hypothesis that treatment versus no treatment of HTN preserves kidney function. The bulk of evidence shows that BP control slows the progression of hypertensive kidney diseases, and control of the BP is not good in Kinshasa.

HTN was also common with type 2 diabetes, which was more frequent than type 1 in the present study. In addition, most patients with DM in this study had poor glucose control. Appropriate management of DM and HTN are important to both the prevention and control of renal disease [23]. Awareness by patient and health care provider will help in this regard, and our study has advanced that awareness in Kinshasa.

Age and duration of DM also influenced the occurrence of CKD in this present study.

Duration of DM is well recognized as an important risk factor for diabetic nephropathy [9]. But duration of HTN did not have a correlation with low eGFR in this survey. That fits well with the fact that in most cases of renal diseases, HTN is the result of, rather than the cause of, the low eGFR. However, we do not know the exact causes of low eGFR in the subjects of this study.

CKD can also result from transmissible diseases such as HIV infection. Our observations show that the prevalence of CKD among HIV positive people is 12%. This value is lower than the 20% found in Uganda [38] and the 27% reported in Soweto [39]. It is on the other hand higher than the 2% [40] and 0.7% [41] described in the USA and in Ethiopia, respectively. This discrepancy of prevalence between these studies could be due to the difference in methodology applied in each survey. It could also reflect an ethnic disparity [42], or even a socio-economic gradient. But, it is hard to determine the true CKD prevalence among HIV population because a validated eGFR method does not exist in HIV subjects.

Among patients with a family history of kidney disease, the proportion of CKD was 8%. In addition, while Ramirez et al [43] showed that FH-KD was a strong determinant of proteinuria, with OR of 2.5, FH-KD in our survey was not associated with proteinuria or with reduction of kidney function. This observation may due to low awareness of CKD and its familial associations by both health workers and the lay population.

Another preventable risk factor for CKD is the use of herbal remedies, which was found in our study in univariate analysis but not in multivariate analysis. Others have reported renal toxicity and other adverse effects of traditional herbal remedies [44]. We can not state which of the remedies are nephrotoxic since studies of their composition are lacking.

Some risk factors for CKD such as smoking or LBW, reported elsewhere [43], were not observed in the present study. However, most of our study population did not have birth certificates or knowledge of their weight at birth, so the risk of LBW is probably not adequately assessed in this study. The reason why tobacco use was not associated with proteinuria remains unclear and deserves further attention.

An important issue that is not resolved by this study is how to reach high-risk individuals who do not attend these clinics as well the problem of payment for antihypertensive and antidiabetic drugs.

We think that our recent model of annual screening for proteinuria and CKD risk factors [45] combining educational message, detection and management of risk factor in general population may contribute partly to reach this first goal.

However, despite their benefits, treatments of HTN and/or DM are costly. This limits their use in SSA, where about half the population lives on less than $ 1 per day [3]. In Kinshasa the annual cost of drugs for optimal antihypertensives such as thiazide diuretics, calcium blocker, or ACE inhibitors, ranges from US $ 100, $ 250, and $ 1000 per patient, respectively, as estimated from a weighted average of wholesale prices for five proprietary products in the market. In this city, it is currently estimated that there are 2.8 million hypertensives [4]. Thus, if all hypertensives were treated with one or more of these drugs we can project that annual cost for these drugs would be between $ 280 million and $ 2.8 billion. If the same drugs are used in combination, the cost of the treatment would be even higher. In patients with DM, the estimated cost per patient of oral antidiabetics and insulin are about $ 200 and $ 350 per year, respectively. If we extrapolate these figures to the current 1.2 million diabetics in Kinshasa [4], the annual cost is over $ 240 million for biguanide-metformin and $ 420 million for insulin. The expenditure in patients with DM and HTN would be even higher. On the assumption that 1% of the hypertensive or diabetic population would evolve to ESRD there would be 28,000 hypertensives and 12,000 diabetics requiring RRT. Hence, we can project the annual cost for RRT in Kinshasa at ~$1.4 billion for peritoneal dialysis or ~2.5 billion for hemodialysis. These estimates for the cost of RRT will be higher if more hypertensives and/or diabetics progress to ESRD. It is clear that long-term dialysis will not be an option for most Africans with renal failure, and it is also likely that the use of anti-hypertensives and better treatment for DM will only reduce, not eliminate, the number of cases of diabetic and hypertensive nephropathy that evolve to ESRD. That said, the increased use of anti-hypertensives and better treatments for DM may reduce non-renal morbidity and mortality, which may make these treatments worth their expense. Indeed, El-Nahas [1] has made a case for the cost-effectiveness of screening for CKD, worldwide, in all populations.

We believe that use of anti-hypertensives and anti-diabetic treatment will reduce morbidity and mortality, but the magnitude of this benefit is not known. Still, in consideration of the combined cost of anti-hypertensive and diabetic treatment, as compared to the expense of cardiovascular disease and chronic dialysis, the cost of anti-hypertensive and diabetic treatment is cheaper. Thus, we need the support of the pharmaceutical industry as well as community support to supply developing countries with necessary renoprotective and cardioprotective drugs.

Furthermore, it is noteworthy that this study population has a very different age distribution from that of the DRC in general, the subjects of this study having an older age than that of the general population of Kinshasa. This could suggest that there will be an increasing number of such at-risk subjects in the near future. Hence, this situation is likely to worsen over the next 20 years if no effective preventive measures are taken.

All health care workers will need to be engaged in this effort. It is impossible that a few nephrologists would be able to implement detection and prevention of CKD for the whole city, let alone the entire DRC. Indeed, in 2004, the DRC counted only 0.11 physicians per 1000 population and 0.52 nurses per 1000 population [46]. Consequently, management of the patients in this country must be by nurses for primary care and by general practitioners for secondary care. Adapting K/DOQI guidelines must account for local conditions and manpower.

### Strength and limitation of our study

The strength of our survey is that it includes patients at-risk for CKD follow-up in multiple centres of primary as well as secondary health care and that we used a random sample and standardised methods of data collection. Already, by doing this study, we have improved awareness of optimal CKD care in traditional health care system of Kinshasa.

Our study has certain limitations. It is a cross-sectional snapshot analysis with 24-hour urinary protein not measured in every patient and serum creatinine measured only once. This may overestimate CKD. It has also a relatively small size, and it relies on the MDRD equation, the validation of which is lacking among African populations as well as in those with HIV. Moreover, the inaccuracy of 24 h urine collection may also over or underestimate CKD. Finally, although all participants were chosen randomly by the local team, it is possible that the choice was made among the more affected patients having needed specialized opinion.

## Conclusion

Despite the above limitations, our findings show the effectiveness of community based targeted health screening program, in identifying significant numbers of persons with CKD previously undiagnosed, and also in finding those with inadequate risk-factor control. This study suggests that there is a great potential for decreasing CKD as well as ESRD incidence and cardiovascular morbidity or mortality by optimizing modifiable risk factors in this high-risk population. It also suggests that the costs of treating those with CKD may be cost-beneficial, by averting the morbidity of cardiovascular disease and also the very high cost of RRT. Furthermore, our study emphasizes the urgent need for increased training of generalist physicians and nurses in CKD prevention programs as well an active collaboration between traditional health care and nephrology services.

The establishment of prevention programs in developing countries is also a political and financial challenge. This challenge could be taken up by creation of international funding assistance as exists for HIV or tuberculosis infection, the goal of which would be to support the program of prevention of CKD, and some of its risk factors such as HTN, DM and obesity.

Finally, further study is needed to improve the precision of these preliminary data and to assess the benefit of antihypertensive such as ACEI on the progression of renal disease in Black Africans. That is because studies in Afro-Americans may probably not be directly applicable to Sub-Saharan Africans and extrapolation must be made with caution.

## Competing interests

The authors declare that they have no competing interests.

## Authors' contributions

EKS designed the study, acquired data, analyzed, interpreted data, drafted and revised the manuscript. EPC interpreted data, drafted and revised the manuscript. CVZ acquired data and revised the manuscript. JMK interpreted data and drafted the manuscript. NMP interpreted data and revised the manuscript. NMN interpreted data and revised the manuscript. EKS had full access to all the study data and assume responsibility for the integrity of the data and the accuracy of the analysis.

All authors read and approved the final manuscript.

## Pre-publication history

The pre-publication history for this paper can be accessed here:

http://www.biomedcentral.com/1471-2369/10/18/prepub

## References

[B1] El Nahas M The global challenge of chronic kidney disease Kidney Int 2005 68 6 2918 2929 10.1111/j.1523-1755.2005.00774.x 16316385

[B2] Hosseinpanah F Kasraei F Nassiri AA Azizi F High prevalence of chronic kidney disease in Iran: a large population-based study BMC Public Health 2009 9 1 44 19183493 10.1186/1471-2458-9-44 PMC2658666

[B3] Arogundade FA Barsoum RS CKD prevention in Sub-Saharan Africa: a call for governmental, nongovernmental, and community support Am J Kidney Dis 2008 51 3 515 523 10.1053/j.ajkd.2007.12.006 18295068

[B4] Sumaili EK Krzesinski JM Zinga CV Cohen EP Delanaye P Munyanga SM Nseka NM Prevalence of chronic kidney disease in Kinshasa: results of a pilot study from the Democratic Republic of Congo Nephrol Dial Transplant 2009 24 1 117 122 10.1093/ndt/gfn469 18715963

[B5] Pakasa NM Sumaili EK The nephrotic syndrome in the Democratic Republic of Congo N Engl J Med 2006 354 10 1085 1086 10.1056/NEJMc052696 16525148

[B6] Krzesinski JM Sumaili EK Cohen E How to tackle the avalanche of chronic kidney disease in Sub-Saharan Africa: the situation in the Democratic Republic of Congo as an example Nephrol Dial Transplant 2007 22 2 332 335 10.1093/ndt/gfl494 17035376

[B7] Dirks JH de Zeeuw D Agarwal SK Atkins RC Correa-Rotter R D'Amico G Bennett PH El Nahas M Valdes RH Kaseje D Prevention of chronic kidney and vascular disease: toward global health equity – the Bellagio 2004 Declaration Kidney Int Suppl 2005 98 S1 6 10.1111/j.1523-1755.2005.09800.x 16108963

[B8] The effect of intensive treatment of diabetes on the development and progression of long-term complications in insulin-dependent diabetes mellitus. The Diabetes Control and Complications Trial Research Group N Engl J Med 1993 329 14 977 986 10.1056/NEJM199309303291401 8366922

[B9] Intensive blood-glucose control with sulphonylureas or insulin compared with conventional treatment and risk of complications in patients with type 2 diabetes (UKPDS 33). UK Prospective Diabetes Study (UKPDS) Group Lancet 1998 352 9131 837 853 10.1016/S0140-6736(98)07019-6 9742976

[B10] Ruggenenti P Perna A Remuzzi G ACE inhibitors to prevent end-stage renal disease: when to start and why possibly never to stop: a post hoc analysis of the REIN trial results. Ramipril Efficacy in Nephropathy J Am Soc Nephrol 2001 12 12 2832 2837 11729254 10.1681/ASN.V12122832

[B11] Brenner BM Cooper ME de Zeeuw D Keane WF Mitch WE Parving HH Remuzzi G Snapinn SM Zhang Z Shahinfar S Effects of losartan on renal and cardiovascular outcomes in patients with type 2 diabetes and nephropathy N Engl J Med 2001 345 12 861 869 10.1056/NEJMoa011161 11565518

[B12] Barsoum RS Chronic kidney disease in the developing world N Engl J Med 2006 354 10 997 999 10.1056/NEJMp058318 16525136

[B13] Boulware LE Jaar BG Tarver-Carr ME Brancati FL Powe NR Screening for proteinuria in US adults: a cost-effectiveness analysis JAMA 2003 290 23 3101 3114 10.1001/jama.290.23.3101 14679273

[B14] de Jong PE Velde M van der Gansevoort RT Zoccali C Screening for chronic kidney disease: where does Europe go? Clin J Am Soc Nephrol 2008 3 2 616 623 10.2215/CJN.04381007 18287253 PMC6631083

[B15] Naicker S Han TM Fabian J HIV/AIDS – dominant player in chronic kidney disease Ethn Dis 2006 16 2 Suppl 2 S2 56 16774012

[B16] Van Deventer HE George JA Paiker JE Becker PJ Katz IJ Estimating Glomerular Filtration Rate in Black South Africans by Use the Modification of Diet in Renal Disease and Cockcroft-Gault Equations Clin Chem 2008 54 7 1197 1202 10.1373/clinchem.2007.099085 18487286

[B17] Ancelle T Statistique épidemiologie 2002 Maloine; FR. Paris

[B18] Chobanian AV Bakris GL Black HR Cushman WC Green LA Izzo JL Jr Jones DW Materson BJ Oparil S Wright JT Jr The Seventh Report of the Joint National Committee on Prevention, Detection, Evaluation, and Treatment of High Blood Pressure: the JNC 7 report JAMA 2003 289 19 2560 2572 10.1001/jama.289.19.2560 12748199

[B19] WHO The problem of overweight and obesity 2000: preventing and managing the global epidemic Report series 894 2000 Wt. Geneva: WHO 537

[B20] Levey AS Coresh J Greene T Stevens LA Zhang YL Hendriksen S Kusek JW Van Lente F Using standardized serum creatinine values in the modification of diet in renal disease study equation for estimating glomerular filtration rate Ann Intern Med 2006 145 4 247 254 16908915 10.7326/0003-4819-145-4-200608150-00004

[B21] K/DOQI clinical practice guidelines for chronic kidney disease 2002: evaluation, classification, and stratification Am J Kidney Dis 2002 39 2 Suppl 1 S1 266 11904577

[B22] Toto RD Kirk KA Coresh J Jones C Appel L Wright J Campese V Olutade B Agodoa L Evaluation of serum creatinine for estimating glomerular filtration rate in African Americans with hypertensive nephrosclerosis: results from the African-American Study of Kidney Disease and Hypertension (AASK) Pilot Study J Am Soc Nephrol 1997 8 2 279 287 9048347 10.1681/ASN.V82279

[B23] Brown WW Peters RM Ohmit SE Keane WF Collins A Chen SC King K Klag MJ Molony DA Flack JM Early detection of kidney disease in community settings: the Kidney Early Evaluation Program (KEEP) Am J Kidney Dis 2003 42 1 22 35 10.1016/S0272-6386(03)00405-0 12830453

[B24] Glassock RJ Winearls C Screening for CKD with eGFR: doubts and dangers Clin J Am Soc Nephrol 2008 3 5 1563 1568 10.2215/CJN.00960208 18667744 PMC4571145

[B25] Hallan SI Dahl K Oien CM Grootendorst DC Aasberg A Holmen J Dekker FW Screening strategies for chronic kidney disease in the general population: follow-up of cross sectional health survey BMJ 2006 333 7577 1047 17062598 10.1136/bmj.39001.657755.BE PMC1647344

[B26] Robert CF Mauris A Bouvier P Rougemont A Proteinuria screening using sulfosalicylic acid: advantages of the method for the monitoring of prenatal consultations in West Africa Soz Praventivmed 1995 40 1 44 49 10.1007/BF01615661 7900435

[B27] Konta T Hao Z Takasaki S Abiko H Ishikawa M Takahashi T Ikeda A Ichikawa K Kato T Kawata S Clinical utility of trace proteinuria for microalbuminuria screening in the general population Clin Exp Nephrol 2007 11 1 51 55 10.1007/s10157-006-0458-z 17384998

[B28] Brantsma AH Bakker SJ de Zeeuw D de Jong PE Gansevoort RT Urinary albumin excretion as a predictor of the development of hypertension in the general population J Am Soc Nephrol 2006 17 2 331 335 16434504 10.1681/ASN.2005111153

[B29] Brantsma AH Bakker SJ Hillege HL de Zeeuw D de Jong PE Gansevoort RT Urinary albumin excretion and its relation with C-reactive protein and the metabolic syndrome in the prediction of type 2 diabetes Diabetes Care 2005 28 10 2525 2530 10.2337/diacare.28.10.2525 16186291

[B30] Iseki K Ikemiya Y Iseki C Takishita S Proteinuria and the risk of developing end-stage renal disease Kidney Int 2003 63 4 1468 1474 10.1046/j.1523-1755.2003.00868.x 12631363

[B31] Kestenbaum B Rudser KD de Boer IH Peralta CA Freid LF Shlipak MG Palmas W Stehman-Breen C Siscovick DS Differences in kidney function and incident hypertension: the multi-ethnic study of atherosclerosis Ann Intern Med 2008 148 501 508 18378946 10.7326/0003-4819-148-7-200804010-00006 PMC3044648

[B32] Couser WG Chronic kidney disease-the promise and the perils J Am Soc Nephrol 2007 18 2803 2805 10.1681/ASN.2007080964 17942947

[B33] Levey AS Atkins R Coresh J Cohen EP Collins AJ Eckardt KU Nahas ME Jaber BL Jadoul M Levin A Chronic kidney disease as a global public health problem: approaches and initiatives – a position statement from Kidney Disease Improving Global Outcomes Kidney Int 2007 72 3 247 259 10.1038/sj.ki.5002343 17568785

[B34] Coresh J Wei GL McQuillan G Brancati FL Levey AS Jones C Klag MJ Prevalence of high blood pressure and elevated serum creatinine level in the United States: findings from the third National Health and Nutrition Examination Survey (1988–1994) Arch Intern Med 2001 161 9 1207 1216 10.1001/archinte.161.9.1207 11343443

[B35] Stevens LA Coresh J Feldman HI Greene T Lash JP Nelson RG Rahman M Deysher AE Zhang YL Schmid CH Evaluation of the modification of diet in renal disease study equation in a large diverse population J Am Soc Nephrol 2007 18 10 2749 2757 10.1681/ASN.2007020199 17855641

[B36] Agodoa LY Jones CA Held PJ End-stage renal disease in the USA: data from the United States Renal Data System Am J Nephrol 1996 16 1 7 16 10.1159/000168965 8719761

[B37] Wright JT Jr Bakris G Greene T Agodoa LY Appel LJ Charleston J Cheek D Douglas-Baltimore JG Gassman J Glassock R Effect of blood pressure lowering and antihypertensive drug class on progression of hypertensive kidney disease: results from the AASK trial JAMA 2002 288 19 2421 2431 10.1001/jama.288.19.2421 12435255

[B38] Peters PJ Moore DM Mermin J Brooks JT Downing R Were W Kigozi A Buchacz K Weidle PJ Antiretroviral therapy improves renal function among HIV-infected Ugandans Kidney Int 2008 74 7 925 929 10.1038/ki.2008.305 18614998

[B39] Fabian J Katz I Gerntholtz T Goetsch S Naicker S Chronic kidney disease in human immunodeficiency virus infection Panminerva Med 2007 49 2 51 66 17625482

[B40] Humphreys MH Human immunodeficiency virus-associated nephropathy. East is east and west is west? Arch Intern Med 1990 150 2 253 255 10.1001/archinte.150.2.253 2405796

[B41] Behar DM Shlush LI Maor C Lorber M Skorecki K Absence of HIV-associated nephropathy in Ethiopians Am J Kidney Dis 2006 47 1 88 94 10.1053/j.ajkd.2005.09.023 16377389

[B42] Kopp JB Winkler C HIV-associated nephropathy in African Americans Kidney Int Suppl 2003 83 S43 49 10.1046/j.1523-1755.63.s83.10.x 12864874

[B43] Ramirez SP McClellan W Port FK Hsu SI Risk factors for proteinuria in a large, multiracial, southeast Asian population J Am Soc Nephrol 2002 13 7 1907 1917 10.1097/01.ASN.0000018406.20282.C8 12089388

[B44] Colson CR De Broe ME Kidney injury from alternative medicines Adv Chronic Kidney Dis 2005 12 3 261 275 10.1016/j.ackd.2005.03.006 16010641

[B45] Sumaili EK Nseka NM Lepira FB Krzesinski JM Makulo JR Bukabau JB Nkoy JB Mokoli VM Longokolo MM Owandjalola JA Screening for proteinuria and chronic kidney disease risk factors in Kinshasa: a World Kidney Day 2007 study Nephron Clin Pract 2008 110 4 c220 228 10.1159/000167869 18974653

[B46] Mills EJ Schabas WA Volmink J Walker R Ford N Katabira E Anema A Joffres M Cahn P Montaner J Should active recruitment of health workers from Sub-Saharan Africa be viewed as a crime? Lancet 2008 371 9613 685 688 10.1016/S0140-6736(08)60308-6 18295027

